# Groundwater level prediction based on a combined intelligence method for the Sifangbei landslide in the Three Gorges Reservoir Area

**DOI:** 10.1038/s41598-022-14037-9

**Published:** 2022-06-30

**Authors:** Taorui Zeng, Kunlong Yin, Hongwei Jiang, Xiepan Liu, Zizheng Guo, Dario Peduto

**Affiliations:** 1 Institute of Geological Survey, China University of Geosciences, Wuhan, 430074 China; 2 Faculty of Engineering, China University of Geosciences, Wuhan, China; 3 School of Environmental and Safety Engineering, Changzhou University, Changzhou, China; 4 School of Civil and Transportation Engineering, Hebei University of Technology, Tianjin, 30041 China; 5 Department of Civil Engineering, University of Salerno, 84084 Salerno, Fisciano Italy

**Keywords:** Environmental sciences, Natural hazards, Engineering

## Abstract

The monitoring and prediction of the groundwater level (GWL) significantly influence the landslide kinematics. Based on the long-term fluctuation characteristics of the GWL and the time lag of triggering factors, a dynamic prediction model of the GWL based on the Maximum information coefficient (MIC) algorithm and the long-term short-term memory (LSTM) model was proposed. The Sifangbei landslide in the Three Gorges Reservoir area (TGRA) in China, wherein eight GWL monitoring sensors were installed in different locations, was taken as a case study. The monitoring data represented that the fluctuation of the GWL has a specific time lag concerning the accumulated rainfall (AR) and the reservoir water level (RWL). In addition, there were spatial differences in the fluctuation of the GWL, which was controlled by the elevation and the micro landform. From January 19, 2015, to March 6, 2017, the measured data were used to set up the predicted models. The MIC algorithm was adopted to calculate the lag time of the GWL, the RWL, and the AR. The LSTM model is a time series prediction algorithm that can transmit historical information. The Gray wolf optimization (GWO) algorithm was used to seek the most suitable hyperparameter of the LSTM model under the specific prediction conditions. The single-factor GWO-LSTM model without considering triggering factors and the support vector machine regression (SVR) model were considered to compare the prediction results. The results indicate that the MIC-GWO-LSTM model reached the highest accuracy and improved the prediction accuracy by considering the factor selection process with the learner training process. The proposed MIC-GWO-LSTM model combines the advantages of each algorithm and effectively constructs the response relationship between the GWL fluctuation and triggering factors; it also provides a new exploration for the GWL prediction, monitoring, and early warning system in the TGRA.

## Introduction

The change of hydraulic condition is one of the crucial factors of landslides. As the most critical hydropower facility of the Yangtze River in China, the Three Gorges reservoir has dramatically changed the geological environment of this area^1^. The length of the TGRA is 637 km; therein more than 2500 landslides activated due to the fluctuation of the RWL^2^. This latter external factor, combined with the seasonal rainfall infiltration, significantly influences the seepage characteristics of landslides and the GWL^3^. The rise of the GWL increases the pore water pressure between the filled soil particles and the rock fissures and decreases the effective stress in the deformation area. The rapid decline of the GWL increases the dynamic water pressure, which accelerates the development of slope instability and deformation. The reduction in effective stresses due to the fluctuation of the GWL, affecting the soil strength and consequently the stability of the slope, causes first-time failures or the reactivation of landside movements^4^. Therefore, the monitoring and prediction of the GWL is an essential part of the landslide hazard analysis^5^. There is a correlation between the landslide displacement velocity and the GWL^6,7^. Van Asch^8^ considered that the increase of the GWL could accelerate the deformation of landslides more than the decrease effect. On the contrary, Zhang^9^ regarded the landslide deformation that occurs in the rapid GWL falling in the TGRA. The GWL changes the material strength at different locations of the landslide, which certainly accelerate the reactivation or sliding of the slope body^6,10,11^. Therefore, the GWL fluctuation analysis is a key part of landslide stability assessment^12^.

The fluctuation of the GWL is complex and depends on external factors such as the surface runoff, the slope morphology^13^, the rainfall intensity^14,15^, the evapotranspiration^16^, the GWL depth^17^, the thickness of unsaturated zone^18^, the natural or anthropogenic drainage features^19^, and the RWL^12^. In this regard, data-driven models can describe the linear relationship between the GWL and triggering factors. Examples provided by Franke^20^ established the regression model between the GWL, the rainfall, and the discharge through the multiple linear regression. Ahn^21^ proposed a second-order difference time series model to predict the groundwater heads. Sutanudjaja^22^ used the soil water index derived from European remote sensing scatter meters to predict the groundwater head in the Rhine–Meuse basin. Cai^23^ adopted the water-table fluctuation method to estimate the groundwater recharge from the water-level and rainfall data. However, the traditional linear model cannot describe the nonlinear hydrological time series. The machine learning time-series model has been widely used in the GWL prediction in recent years. Tsanis^24^ used the feed-forward neural network to predict the GWL. Yoon^25^ adopted the artificial neural network and the SVR model to analyze the GWL in a coastal aquifer and selected the past GWL, precipitation, and tide level as the triggering factors. Krakac^4,26^ established a random forest model of multiple triggering factors with the GWL. Cao^12^ proposed a time-series prediction model for the RWL, the RA, and the GWL according to the peculiar hydrogeological conditions in the TGRA.

The single machine learning model is widely used in time-series prediction with the advantages of simple implementation and fast operation speed^27^, such as the back-propagation neural network model (BPNN)^28^, kernel extreme learning machine (KELM)^29,30^, least-squares SVR^31^, Volterra filter model^32^, Kalman filter method^33^, SVR^34^ and so on. However, the complex nonlinear time series will reduce the prediction accuracy of a single model. At present, the combined intelligence method of two (e.g., one for optimization and one for prediction) or more algorithms has become a vital point of the prediction model. The prediction performance of this combined model is always better than that of a single model^35^. The prediction models can improve prediction accuracy and operation efficiency by optimizing their hyperparameters. For example, the BPNN model usually needs to optimize its initial weight and threshold^36^. The SVR and KELM models need to find the optimal penalty factor and kernel function parameter^37,38^. At present, the metaheuristic search algorithms were widely applied to optimize the hyperparameters, such as the particle swarm optimization (PSO)-SVR model^39–42^, the PSO-ELM model^35^, the GWO-ELM model^38,43^, the GWO-BP model^36^, the ant colony optimization (ACO)-SVR model^37^, the genetic algorithm (GA)-SVR model^12^, etc. However, there are still two limits to practical application: (1) Different triggering factors have different contributions to the prediction model. The redundant features can affect the prediction accuracy. (2) These models consider both the factors and output data at different time points as independent vectors. To overcome these two issues, the MIC algorithm was proposed to determine the crucial triggering factors in this paper. The MIC measured the linear and nonlinear relations between different data variables, and reflected their non-functional dependence^44^. The LSTM algorithm was applied to predict the monitoring data as it can reflect the long-term dependence between the time step and data series^45–47^. Therefore, this paper adopted the MIC algorithm to sort the redundant features and the GWO-LSTM model to predict the GWL of landslides. Specifically, the main objectives include: (i) using a MIC algorithm to calculate the lag time of the GWL, the RWL, and the AR of the landslide; (ii) applying a MIC-GWO-LSTM model to update and predict each sequence of monitoring data; and (iii) comparing the predicted and measured GWL for the model validation.

## Materials

### Geological and kinematic features

**Figure 1:**
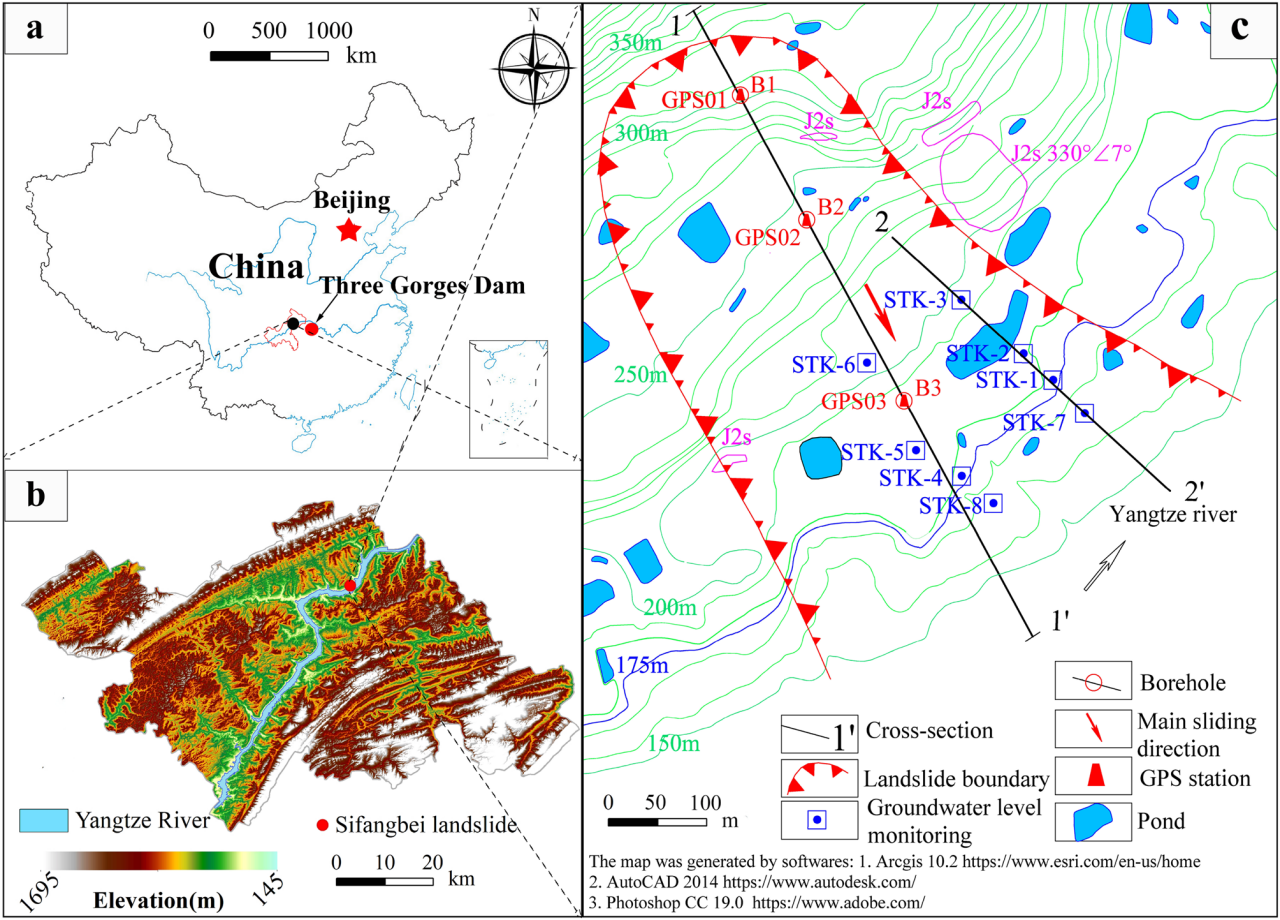
(**a**) Location of the study area; (**b**) location of Sifangbei landslide; (**c**) topographical map of the Sifangbei landslide, with the location of the monitoring network.

**Figure 2:**
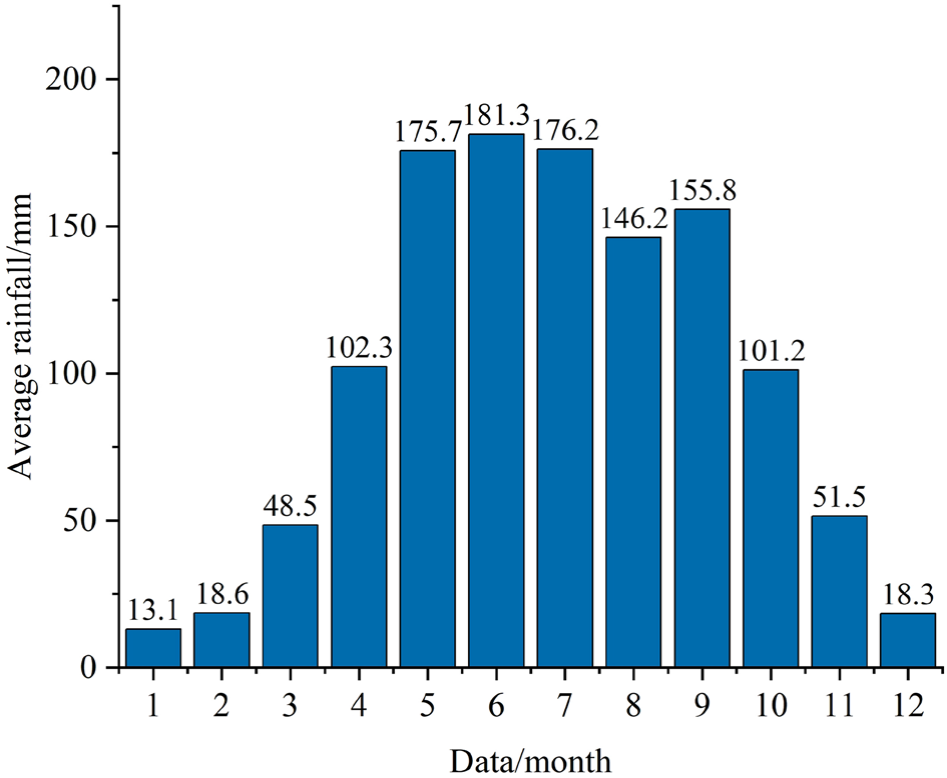
Average monthly rainfall in Wanzhou District (1960–2015).

The Sifangbei landslide is located in the Wanzhou District, Chongqing (108° 29′ 18.65″ E, 30° 51′ 45.69″ N), which is on the north bank of the Yangtze River (Fig. 1). The Wanzhou District has abundant rainfall with a typical subtropical humid monsoon climate. According to the monitoring data of the Geo-Environmental Monitoring Office, the annual average temperature in Wanzhou District is 18.1 °C, the minimum temperature is − 3.7 °C, the maximum temperature is 42.1 °C, the relative humidity is 80%, the frost-free period is 334 days, and the annual sunshine hours are 1484.4 h. The average annual rainfall in Wanzhou is 1202.8 mm (1960–2015). The rainfall mainly concentrates from May to September every year, accounting for about 70% of the annual rainfall (Fig. 2). The maximum average monthly rainfall is 203.1 mm in July. The historical maximum annual rainfall was 1635.2 mm (1981), the maximum monthly rainfall was 711.8 mm (July 1982), and the maximum daily rainfall was 243.31 mm (July 16, 1982).

The Sifangbei landslide has an armchair shape in plain view with an elevation between 110 and 325 m. It has an estimated volume of 9.03 × 10^6^ m^3^ and covers an area of 3.612 × 10^5^ m^2^, with a length of 840 m and a width of 430 m. The sliding body depth (Fig. 2) varies from 10.3 to 28.9 m, and the average bedrock depth is 22 m. The surface water network of Sifangbei landslide is relatively developed, mainly including a water pond, farmland, and gully. The pond on the landslide has water all year round, with a total area of about 1.2 × 10^4^m^2^. According to the occurrence characteristics, the groundwater of the Sifangbei landslide can be divided into bedrock fissure water and loose pore water. The pore water is mainly distributed in the Quaternary deposits and rubble soil layer, mainly supplied by atmospheric precipitation and surface water, and discharged into the river along the bedrock surface.

**Figure 3:**
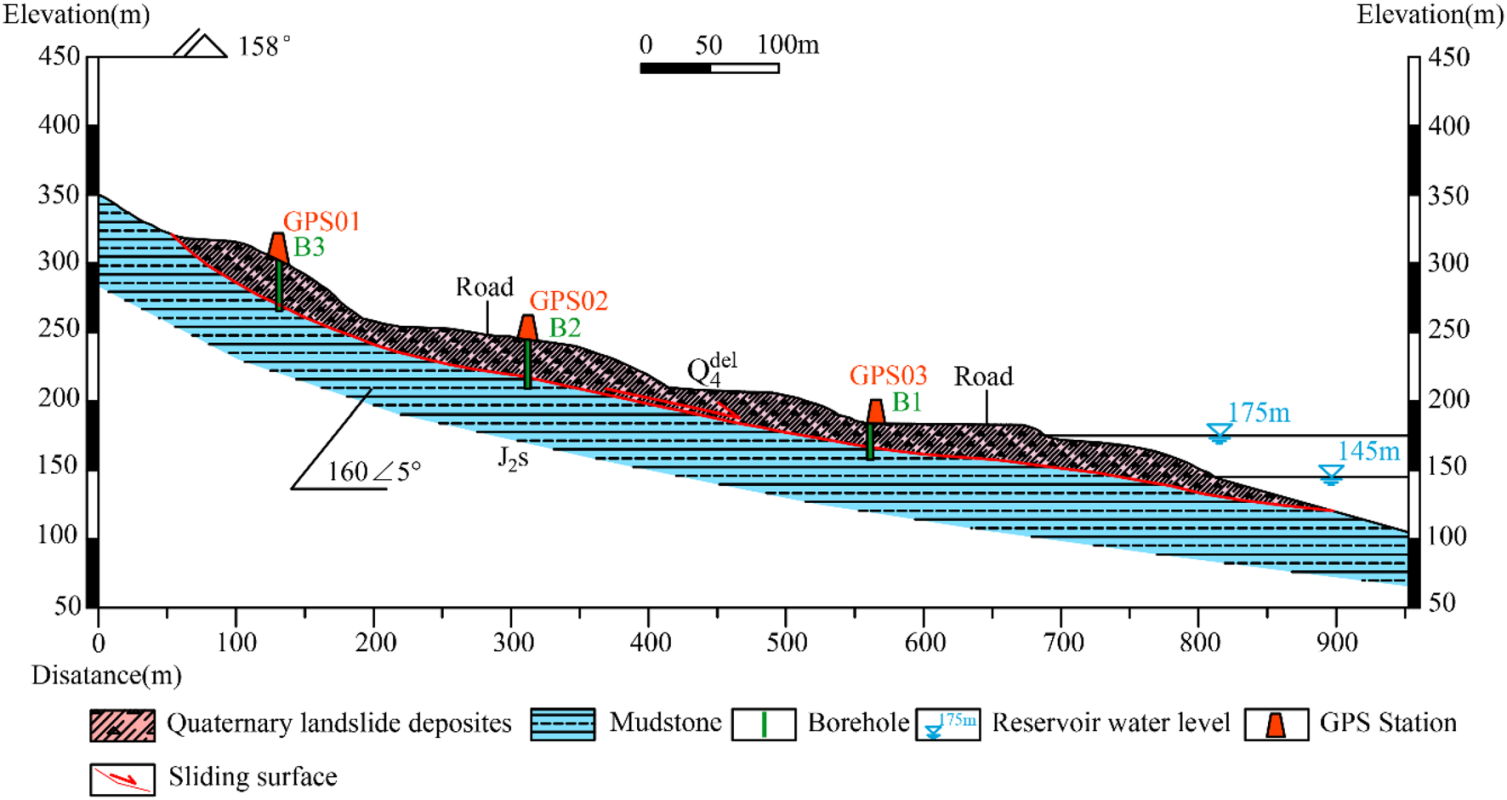
Engineering geological profile 1–1’ of the Sifangbei landslide.

The engineering geological profile of the Sifangbei landslide is stepped form with a main sliding direction in 160° (Fig. 3). There are two-level platforms developed in the landslide area, the central platform is 220 m long, 450 m wide, and 200 m high. The sliding surface in correspondence of the landslide toe is below the water level of the Yangtze River. The surface layer of the landslide consists of thick quaternary silty clay mixed with broken rocks. The underlying layer is typical near-horizontal Jurassic strata in Wanzhou District. The occurrence of the strata is 160°∠5°.

**Figure 4:**
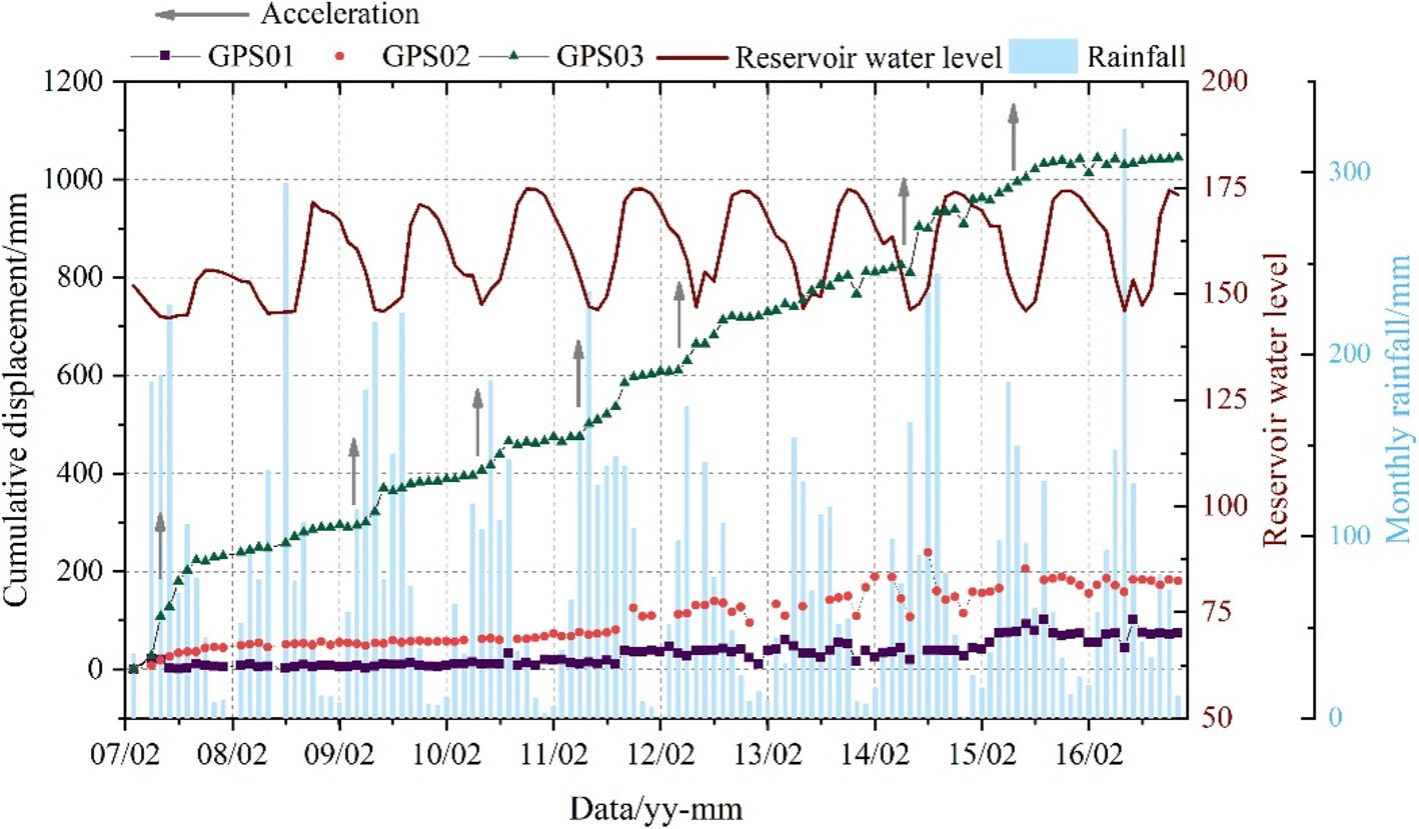
Monitoring data: rainfall, reservoir water level, and cumulative displacement of Sifangbei landslide.

The initial monitoring network of the Sifangbei landslide was established in March 2007, which includes 3 GPS stations (Fig. 1). The Sifangbei landslide began to deform in June 2001. In May 2007, the deformation of the Sifangbei landslide was accelerated due to the first water storage of Three Gorges Reservoir to 156 m, which caused many through cracks and secondary collapses in the middle-front area of the landslide. Based on the surface displacement data collected from March 2007 to April 2016 (Fig. 4), the cumulative displacement of GPS03 is the largest, followed by GPS02 and GPS01. The rapid increase of displacement mainly occurred during the rapid decline period of RWL and the month of heavy rainfall. Accordingly, it can be concluded that the deformation of the Sifangbei landslide is mainly affected by RWL and rainfall.

### In-situ monitoring and data acquisition

Members of the research group started a field investigation in Sifangbei in July 2014 and established a hydrological monitoring system in 2015. Data for the present study are collected from the Sifangbei landslide hydrological monitoring system. The monitoring system (see Fig. 1) consists of six Semi-automatic GWL holes (STK1–STK6) and two automatic GWL holes (STK7, STK8). The semi-automatic GWL holes are measured by a MicroDiver probe, which can store up to 48,000 groups of data, with an accuracy of ± 0.05%, and a regular working temperature of 0–40 °C. The automatic GWL holes are a voltage-type water level gauge, with an accuracy of ± 0.25%. Both holes measure GWL once a day. After collecting and saving the device record data, the GWL can be obtained through the calculation formula:

1 $$H = \frac{h}{100} - h_{s} { + }H_{s} - d$$

where *H* is the groundwater level (m), *h* is the device record data (cm), *h*_*s*_ is the height of the water column at standard atmospheric pressure (m) and it is equal to 10.336 m, *H*_*s*_ is the elevation of the monitoring holes (m), *d* is the distance between the MicroDiver probe and the monitoring hole.

**Figure 5:**
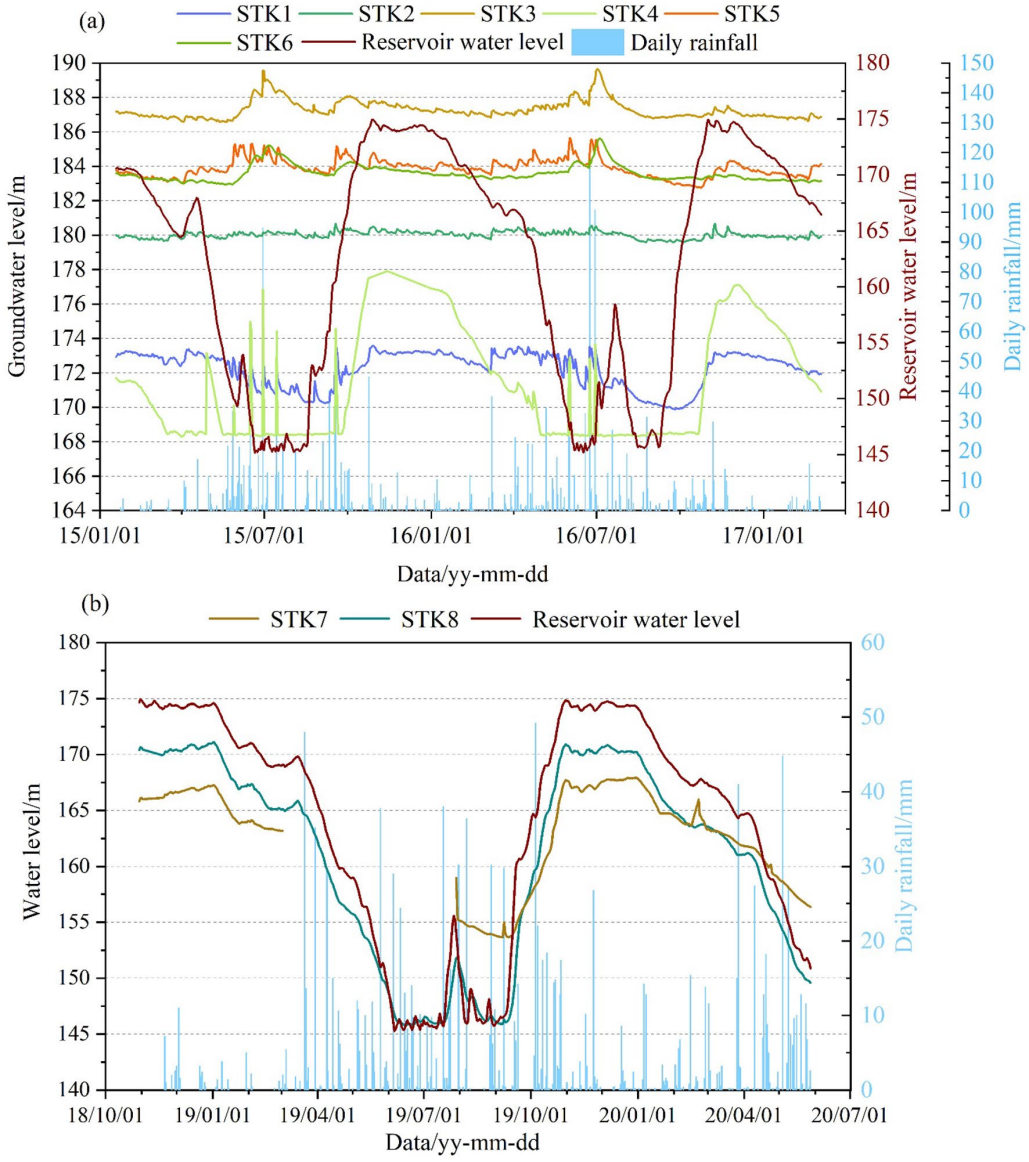
Monitoring plots of the groundwater level holes: (**a**) the semi-automatic groundwater level holes; (**b**) the automatic groundwater level holes.

The GWL of the Sifangbei landslide has been monitored since 2015, including the STK1–STK6 from January 19, 2015, and the STK7, and STK8 from October 30, 2018. Considering the missing data and maintenance of monitoring points, periods with more complete data were selected for analysis. The data of the semi-automatic GWL holes were selected from January 19, 2015, to March 6, 2017, and data of the automatic GWL holes were selected from October 30, 2018, to May 28, 2020 (Fig. 5).

**Figure 6:**
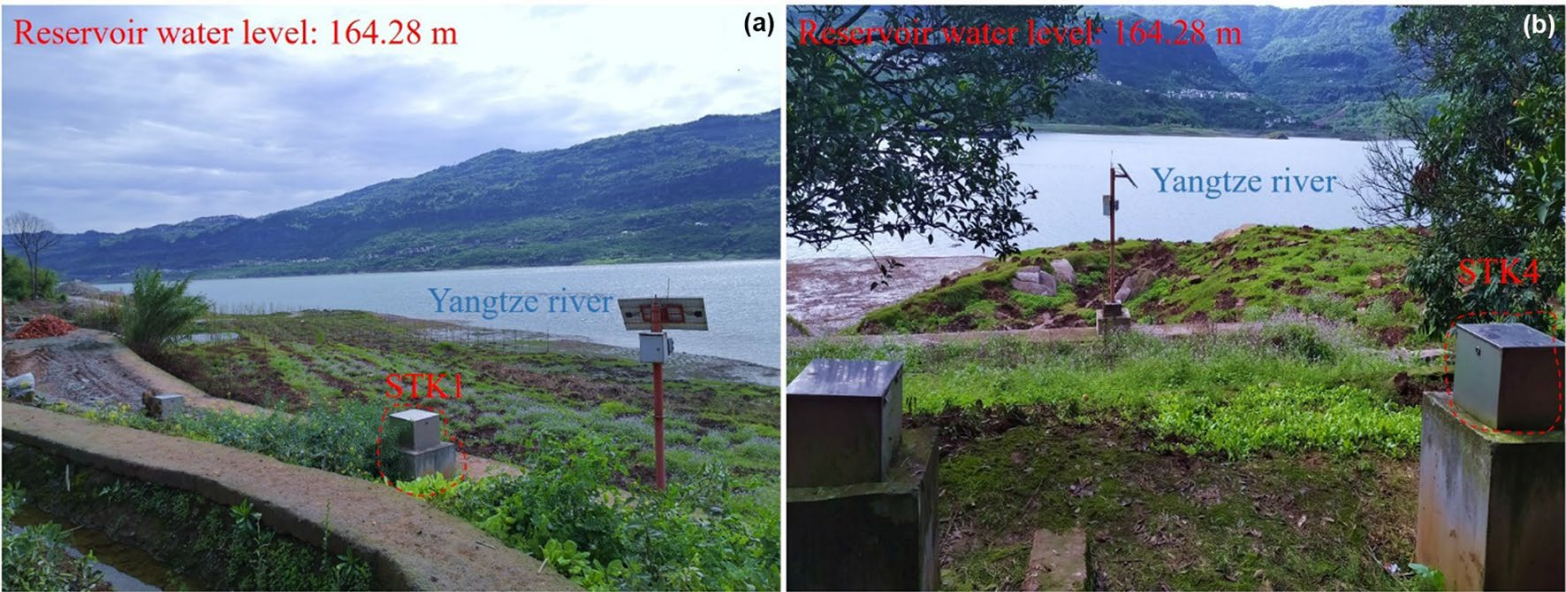
Hydrological monitoring hole of 175 m water level. (**a**) The STK1 monitoring hole; (**b**) the STK4 monitoring hole.

The GWL monitoring holes can be divided into four groups (Table 1). The distance of each group from the Yangtze River is shown in Fig. 1. According to different groups, the GWL of Sifangbei can be analyzed:

1. The STK7 and STK8 are located in the fluctuation range of RWL (175–145 m). The fluctuation of monitoring data of these two points is positively correlated with the RWL, and almost at the same value. The monitoring point is the underwater part of each year, so it is less affected by rainfall.
2. The elevation of the STK1 and STK4 is 175 m and they are close to the Yangtze River. The fluctuation of the GWL is affected by the RWL. The GWL of STK1 ranges from 169.69 to 173.59 m as a gentle slope behind the monitoring hole (Fig. 6a) and also shows a short-term fluctuation during the rainy season. The GWL of STK4 ranges from 168.29 to 177.88 m, mainly due to the loose surface soil and high permeability coefficient caused by the planting of fruit trees (Fig. 6b). The increase of hydrodynamic pressure and seepage pressure accelerates the deformation of the landslide front area.
3. The STK2 and STK5 are located on the first-level platform of the Sifangbei landslide. The STK2 is located near farmlands and ponds and is mainly affected by human agricultural activities. The GWL of STK2 is always maintained at about 180 m in the whole process of RWL regulation. The GWL of STK5, mainly affected by rainfall, is far away from the Yangtze River and mainly fluctuates in the rainy season.
4. The STK3 and STK6 are the farthest monitoring points from the Yangtze River and are mainly controlled by rainfall. The platform on the slope collected water in the rainy season, resulting in the rapid rise of the GWL. After the rainy season, the groundwater was discharged along the slope. The primary performance of these two points represents a periodic fluctuation within the year, which is a rapid rise followed by a rapid fall.

**Table 1:** Grouping of groundwater level monitoring holes.

Group	Monitoring holes	Correlation with triggering factors	
		Rainfall	Reservoir water level
1	STK7	Low	High
	STK8	Low	High
2	STK1	High	High
	STK4	Medium	High
3	STK2	Low	Low
	STK5	High	Low
4	STK3	Medium	Low
	STK6	Medium	Low

**Figure 7:**
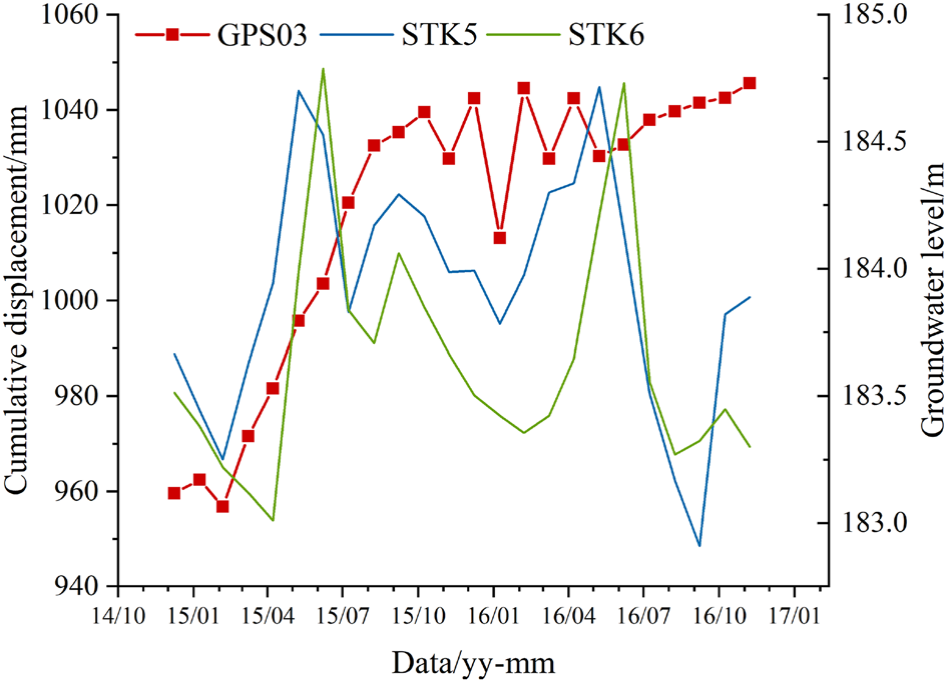
Monitoring data comparison of GPS03, STK5, and STK6.

The GPS03, STK5, and STK6 hydrological holes (relatively close) were selected to analyze the relationship between the horizontal surface displacement and the GWL. As shown in Fig. 7, there is a relatively good fitting between the surface displacement and the GWL. The fluctuation of the GWL causes the fluctuation of cumulative displacements. The adjacent elevation hydrological hole (STK5) can better reflect the fluctuation in surface displacement (GPS03). The rapid fluctuation of the GWL causes an increase in displacement. Especially from February 2015 to September 2015, the cumulative displacement increased by 77.1 mm. Therefore, the monitoring and analysis of groundwater levels can reflect the characteristics of landslide deformation.

Table 1 represents the qualitative correlation of different monitoring points with rainfall and RWL. At the front edge of the landslide, the fluctuation of the GWL is mainly affected by RWL, and at a distance from the Yangtze River, it is mainly affected by rainfall. The STK1 monitoring hole, which the RWL and rainfall influence, was used to establish the prediction model for the present study.

## Methodology

### Time series analysis

In the TGRA, the GWL in reservoir landslide is related to the RWL and the rainfall. According to the data of the RWL, the AR, and the GWL, the time-series relationship is established as follows:

2 $$g_{t} = f(x_{1} ,x_{2} , \ldots ,x_{n} ,y_{1} ,y_{2} , \ldots ,y_{i} )$$

where *g*_*t*_ is the GWL at time *t*, *f* is the prediction model, *x*_*n*_ is RWL factors, *y*_*i*_ is rainfall factors.

### Long short-term memory neural networks

**Figure 8:**
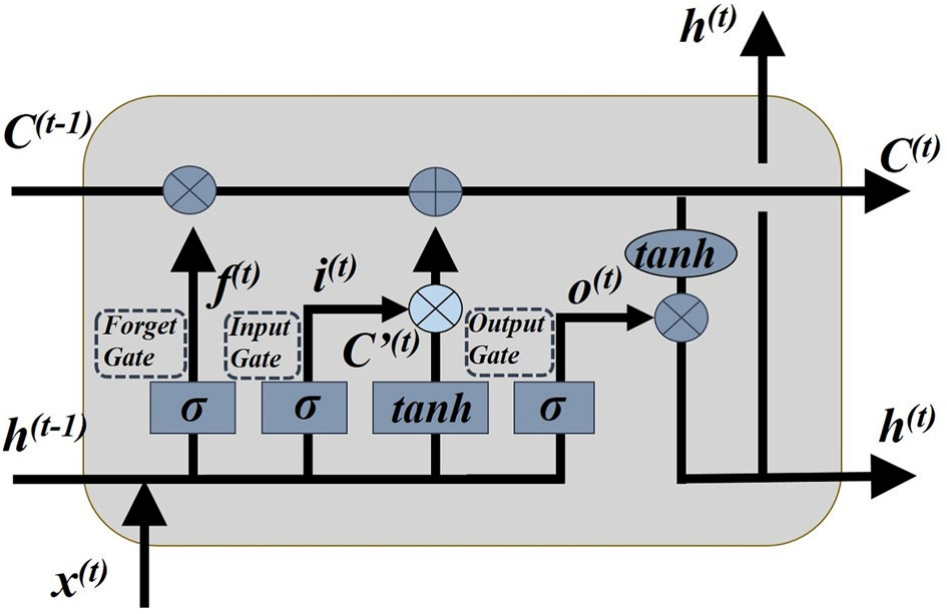
The LSTM module contains four interacting layers.

The input-implicit-output layer is fully connected to traditional neural networks. The points between the sequences are not connected. Therefore, the traditional neural network is not suitable for time series prediction^48^. The recurrent neural network (RNN) records the previous information and applies it to the current output calculation, which allows for feedback in the networks^49^. The LSTM neural networks are a special type of RNN, which can effectively solve the problem of "gradient disappearance" or "gradient explosion"^50^. The LSTM ensures data discovery and long-term memory through the forgetting gate, input gate, and output gate. The three gate functions provide a good nonlinear control mechanism for the input and deletion of control information (Fig. 8). The hidden vector (*h*^*(t)*^) in the LSTM model can be obtained as follows:

3 $$\left\{ {\begin{array}{*{20}l} {f^{(t)} = \sigma \left( {W_{f} h^{{\left( {t - 1} \right)}} + U_{f} x^{(t)} + b_{f} } \right)} \hfill \\ {i^{(t)} = \sigma \left( {W_{i} h^{{\left( {t - 1} \right)}} + U_{i} x^{(t)} + b_{i} } \right)} \hfill \\ {c^{^{\prime}(t)} = \tanh \left( {W_{c} h^{(t - 1)} + U_{c} x^{(t)} + b_{c} } \right)} \hfill \\ {C^{(t)} = C^{(t - 1)} \odot f^{(t)} + i^{(t)} \odot c^{^{\prime}(t)} } \hfill \\ {o^{(t)} = \sigma \left( {W_{o} h^{(t - 1)} + U_{o} x^{(t)} + b_{o} } \right)} \hfill \\ {h^{(t)} = o^{(t)} \odot \tanh \left( {C^{(t)} } \right)} \hfill \\ {y^{(t)} = \sigma \left( {W_{y} h^{(t)} + b_{y} } \right)} \hfill \\ \end{array} } \right.$$

where *f*^(t)^, *i*^(t)^, *o*^(t)^and *c*^’(t)^are the values of the forget gate, the input gate, forget gate, the output gate, and the memory cell in the memory block; *W*_(*f*, *i*,*c*,*o*)_ is the weight of *h*^(t-1)^; *U*_(*f*, *i*,*c*,*o*)_ is the weight of input data *x*^(t)^; *b*_*f*_, *b*_*c*_, *b*_*i*_ and *b*_*o*_ are their corresponding bias values; *σ* is the sigmoid function, tanh is the hyperbolic tangent function; *C*^(t)^ is the updated value of the cell state; *y*^(t)^ is the output value at time t. After forward propagation, BPTT (backpropagation through time) algorithm is used to transfer the accumulated error back from the last time, calculate the gradient of error corresponding parameters. Finally, the weights and thresholds are updated by the stochastic gradient descent algorithm.

### Grey wolf optimizer

**Figure 9:**
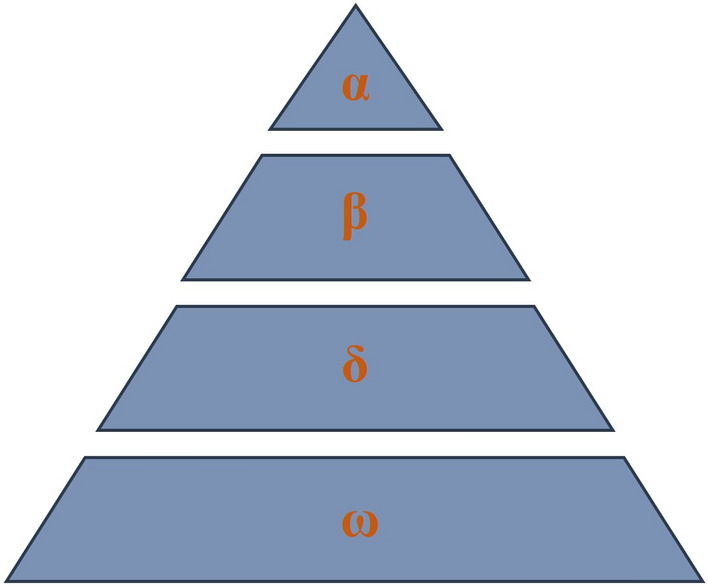
Hierarchy of grey wolf (dominance decreases from the top-down).

The GWO is a novel meta-heuristic algorithm with strong search ability that imitates the leadership and hunting mechanism of gray wolves in nature^51^. It uses three wolf heads (α wolf, β wolf, and δ Wolf) to determine the fitness value, while other wolves calculate the distance between them and their prey according to the location of their prey^51^ (Fig. 9).

(1) According to the level of the wolf group, the optimal solution is regarded as *α* wolf, the second and third optimal solutions are *β* wolf and *δ* wolf respectively, and the other candidate solutions are *ω* wolf.

(2) The distance $\vec{D}$ between the prey and the grey wolves is determined before preying:

4 $$\vec{D} = \left| {\vec{C} \cdot \vec{X}_{P} (t) - \vec{X}(t)} \right|$$

where $\vec{C}$ is the swing factor, $\vec{C}{ = }2\vec{r}$; $\vec{r}$ is the random vector,$\vec{r} = random[0,1][0,1]$; $\vec{X}_{P}$(t) is the position vector of the prey of the t_th_ generation of grey wolves; $\vec{X}(t)$ is the position vector of the t_th_ generation of grey wolves.

(3) The distance between the gray wolf and its prey is shortened by iterative updating:

5 $$\vec{X}(t + 1) = \vec{X}_{P} (t) - \vec{A} \cdot \vec{D}$$

6 $$\vec{A}{ = }2\vec{a} \cdot \vec{r} - \vec{a}$$

where $\vec{A}$ is the convergence factor; $\vec{a}$ is the weight factor, and the initial value is 2, which decreases to 0 as the number of iterations increases. A grey wolf in the position of (X, Y) can update its position according to the position of the prey (X*, Y*). Different places around the best agent can be reached concerning the current position by adjusting the value of $\vec{A}$ and $\vec{C}$ vectors.

**Figure 10:**
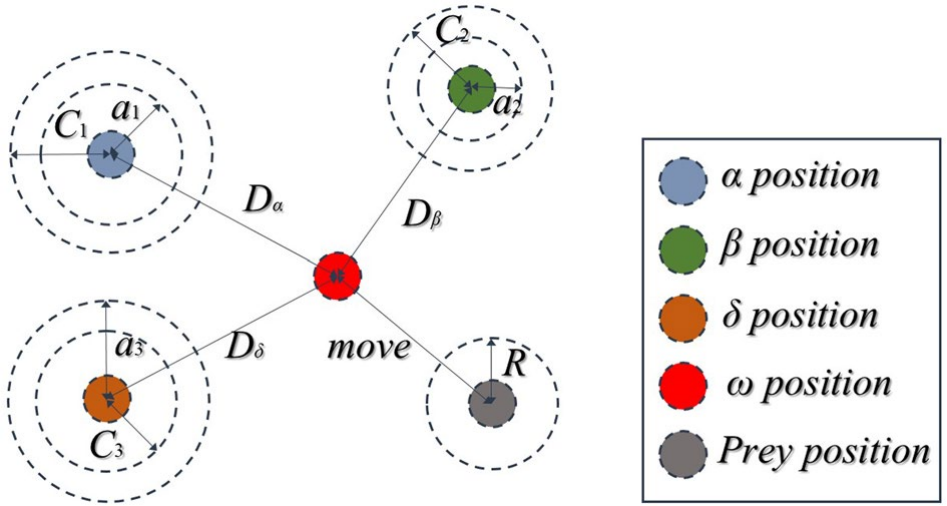
Position updating in GWO.

(4) In the abstract search space, the *ω* wolf moves closer to the prey according to the position of the three wolves (Fig. 10). The final position is determined by the random position in the circle defined by *α*, *β,* and *δ* in the search space. The distance between the other grey wolves and these three wolves in the *t* the generation can be obtained according to the following formula:

7 $$\vec{D}_{\alpha } = \left| {\vec{C}_{1} \cdot \vec{X}_{\alpha } - \vec{X}} \right|,\vec{D}_{\beta } = \left| {\vec{C}_{2} \cdot \vec{X}_{\beta } - \vec{X}} \right|,\vec{D}_{\delta } = \left| {\vec{C}_{3} \cdot \vec{X}_{\delta } - \vec{X}} \right|$$

8 $$\vec{X}_{1} = \vec{X}_{\alpha } - \vec{A}_{1} \cdot (\vec{D}_{a} ),\vec{X}_{2} = \vec{X}_{\beta } - \vec{A}_{2} \cdot (\vec{D}_{\beta } ),\vec{X}_{3} = \vec{X}_{\delta } - \vec{A}_{3} \cdot (\vec{D}_{\delta } )$$

9 $$\vec{X}(t + 1) = \frac{{\vec{X}_{1} + \vec{X}_{2} + \vec{X}_{3} }}{3}$$

**Figure 11:**
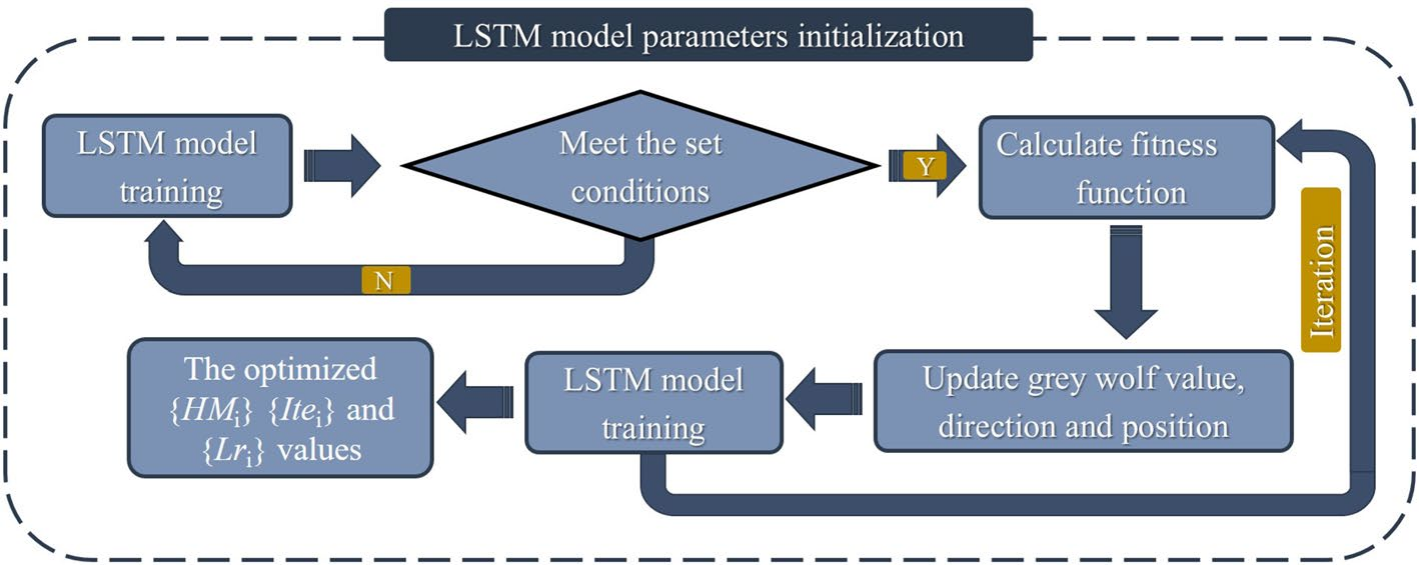
Analysis flowchart of the GWO-LSTM prediction model.

The number of hidden layer neurons, the number of iterations, and the learning rate have a significant impact on the fitting and prediction ability of the LSTM model. Therefore, the GWO algorithm was introduced to optimize the hyperparameter of the LSTM model in landslide displacement prediction. The flowchart is depicted in Fig. 11. The optimal hyperparameters of the GWO-LSTM model were obtained by using the training set and the testing set. Considering the continuity and historical memory of time series, the training set and the testing set were combined as the new training set, and they get the prediction results compared with the verification set, which verifies the prediction accuracy and generalization ability of the model.

### Maximal information coefficient

The MIC is a new variable correlation analysis method proposed by D. n. Reshef et al.^52^ in 2011 based on mutual information (MI). This method can not only find the linear functional relationship between variables but also find the nonlinear functional relationship (exponential, periodic, etc.)^44^. The main steps of the algorithm are as follows:

1. Supposed there is a connection between two variables *V*_1_ = {*v*_1_(*i*)}, *i* = 1,2,…,*n* and *V*_2_ =  = {*v*_2_(*i*)},*i* = 1,2,…,*n*. D is the set of ordered pairs {*v*_1_(i),*v*_2_(i)},*i* = 1,2,…,*n*. Using grid G_1_ (*x*_1_ × *y*_1_) to divide *V*_1_ sample points into *x*_1_ and *V*_2_ sample points into *y*_1_, some cells are allowed to be empty sets.
2. The characteristic matrix $M(D)_{{x_{1} ,y_{1} }}$ can be obtained from the maximum mutual information value $\max I(D|_{{G_{1} }} )$ as follows:
  10 $$M(D)_{{x_{1} ,y_{1} }} = \frac{{\max I(D|_{{G_{1} }} )}}{{\ln \min (x_{1} ,y_{1} )}} = {{\max \left( {\sum\limits_{i = 1}^{{x_{1} }} {\sum\limits_{j = 1}^{{y_{1} }} {\frac{{n_{ij} }}{N}\ln \frac{{n_{ij} }}{N} - \sum\limits_{i = 1}^{{x_{1} }} {\frac{{\sum\limits_{j = 1}^{{y_{1} }} {n_{ij} } }}{N}\ln \frac{{\sum\limits_{j = 1}^{{y_{1} }} {n_{ij} } }}{N}} } } - \sum\limits_{i = 1}^{{y_{1} }} {\frac{{\sum\limits_{j = 1}^{{x_{1} }} {n_{ij} } }}{N}\ln \frac{{\sum\limits_{j = 1}^{{x_{1} }} {n_{ij} } }}{N}} } \right)} \mathord{\left/ {\vphantom {{\max \left( {\sum\limits_{i = 1}^{{x_{1} }} {\sum\limits_{j = 1}^{{y_{1} }} {\frac{{n_{ij} }}{N}\ln \frac{{n_{ij} }}{N} - \sum\limits_{i = 1}^{{x_{1} }} {\frac{{\sum\limits_{j = 1}^{{y_{1} }} {n_{ij} } }}{N}\ln \frac{{\sum\limits_{j = 1}^{{y_{1} }} {n_{ij} } }}{N}} } } - \sum\limits_{i = 1}^{{y_{1} }} {\frac{{\sum\limits_{j = 1}^{{x_{1} }} {n_{ij} } }}{N}\ln \frac{{\sum\limits_{j = 1}^{{x_{1} }} {n_{ij} } }}{N}} } \right)} {\ln \min (x_{1} ,y_{1} )}}} \right. \kern-\nulldelimiterspace} {\ln \min (x_{1} ,y_{1} )}}$$

where $D|_{{G_{1} }}$ is the probability mass distribution function of all cells in grid *G*_1_; *n*_*ij*_ is the sample point in column *i* of row *j* in grid *G*_1_, *N* is the total number of samples.

Because different grid *G* will lead to different $D|_{G}$, the global optimal grid *G*_0_ (*x*_0_ × *y*_0_) is determined by an exhaustive search of the characteristic matrix. The maximum information coefficients of variables *V*_1_ and *V*_2_ are as follows:

11 $$MIC(D) = \mathop {\max }\limits_{xy < B(N)} \left\{ {M(D)_{x,y} } \right\} = \max \frac{{\max I(D|_{G} )}}{\ln \min (x,y)} = M(D)_{{x_{0} ,y_{0} }}$$

where *B* (*N*) is the maximum grid area for searching. In general, The MIC algorithm is essentially a normalized maximum mutual information, and its value range is [0, 1]. For two independent variables, the MIC tends to be 0; for two variables with noise-free function relationships, the MIC tends to 1. To verify the accuracy of prediction, the *RMSE*, and the *R*^2^ was used to evaluate the prediction accuracy^53^.

## Results

### Correlation analysis of triggering factors

**Figure 12:**
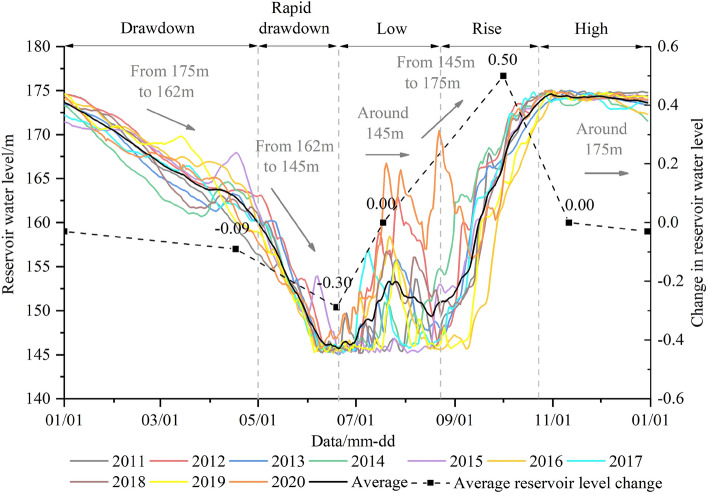
Annual variation of reservoir water level.

Since 2009, the main fluctuation range of water level in the TGRA has been 145–175 m. According to the data collected by Wanzhou hydrological station, the maximum Yangtze River annual runoff is 4518 × 10^8^ m^3^, with a minimum annual runoff of 2573 × 10^8^ m^3^ and an average annual runoff of 3705 × 10^8^ m^3^. The annual water level variation has five stages^54^ (Fig. 12): (1) slow drawdown from 175 to 162 m (January–May); (2) rapid drawdown from 162 to 145 m (May- middle June); (3) around 145 m (middle June–late August); (4) rise from 145 to 175 m (late August-October); (5) around 175 m (November–December). Accordingly, the hydrogeological conditions of the TGRA present different characteristics over the year^30^.

The long-term fluctuation of the GWL is related to the RWL, while the short-term fluctuation is affected by AR. The GWL represents different degrees of time lag with the change of external conditions and soil permeability. It can be concluded that the response time lag of the STK1 monitoring GWL to the RWL is short from the geographical location and monitoring data.

**Figure 13:**
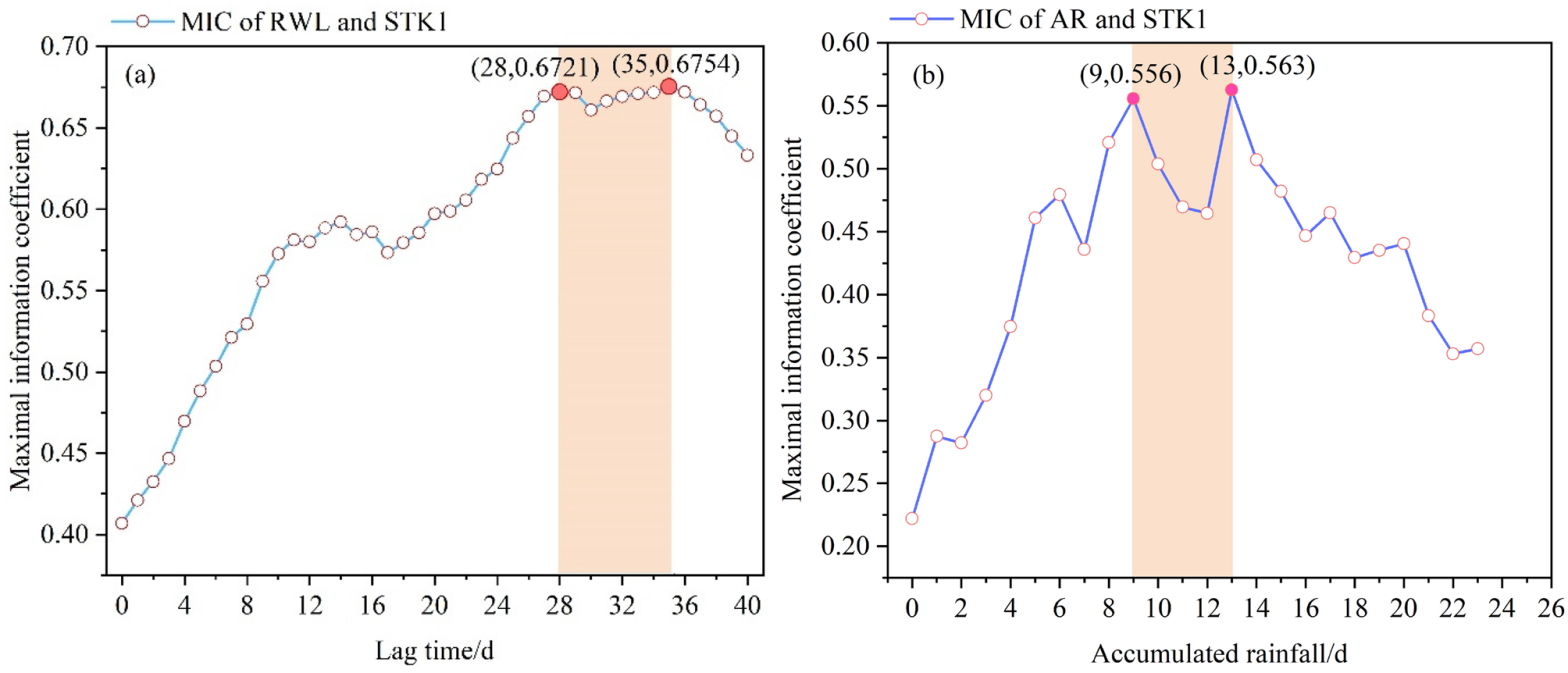
The MIC of the GWL and triggering factors: (**a**) the reservoir water level; (**b**) the accumulated rainfall.

**Figure 14:**
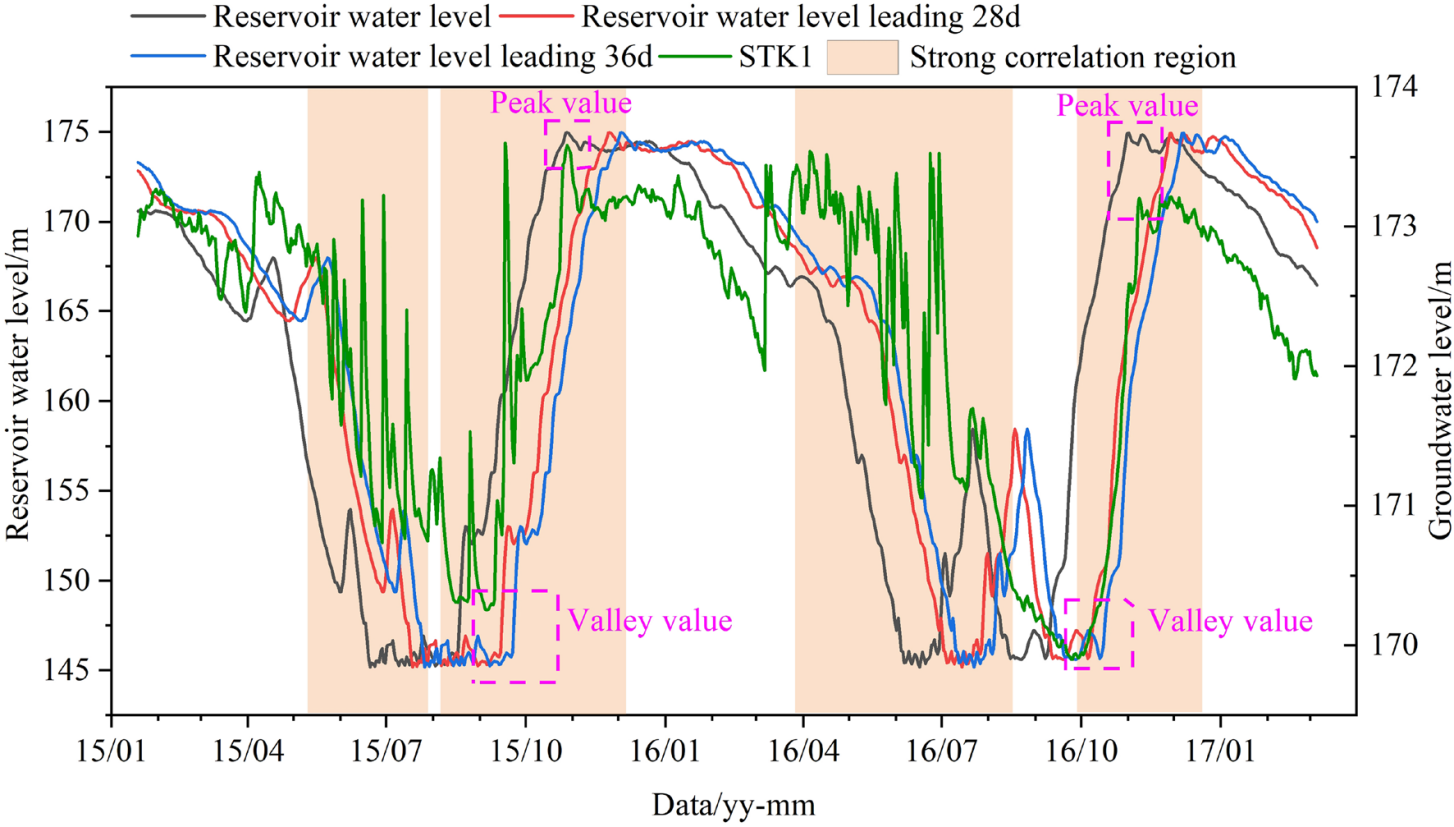
The curve chart of leading reservoir water level and groundwater level in STK1.

The most crucial step of the GWL prediction is determining the relevant triggering factors. The MIC algorithm was adopted to determine the correlation between the GWL and triggering factors. Figure 13a shows that the correlation is the largest when the RWL leads GWL 28–35 days. The curve chart of the RWL and the GWL (Fig. 14) shows that the overall trend is consistent with the leading RWL; the peak value of the GWL is the same as that of the current RWL, the valley value periodic change is consistent with that of the leading RWL.

**Figure 15:**
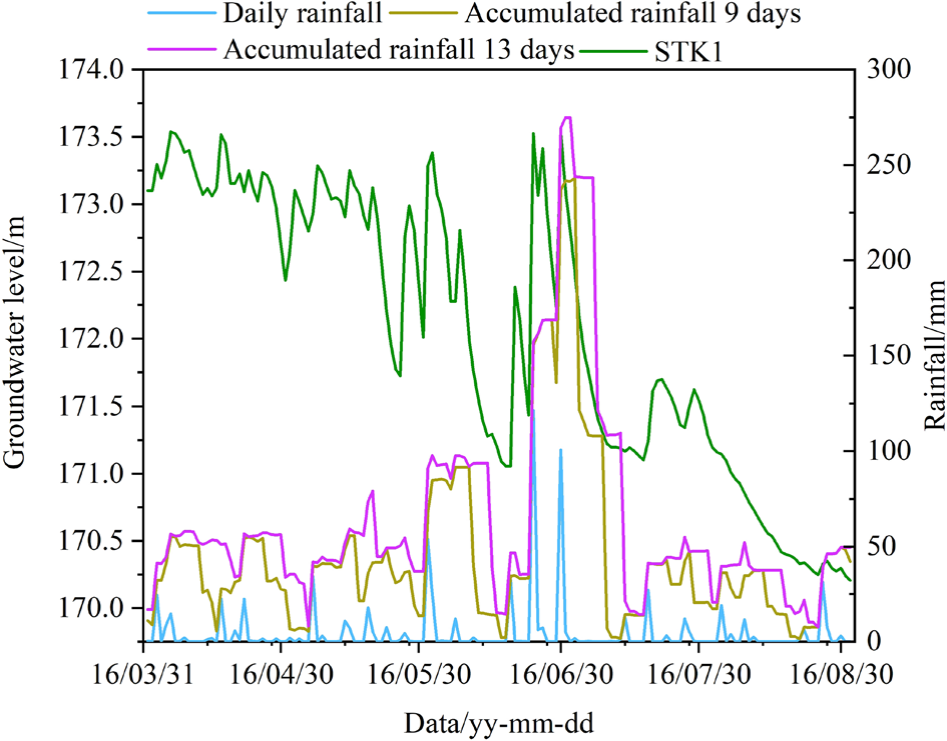
The curve chart of accumulated rainfall and groundwater level in STK1.

The short-term fluctuation of the GWL is affected by the AR. The AR and the GWL data from April 1, 2016, to September 1, 2016, were selected for correlation analysis (Fig. 13b). In particular, the most significant correlation was recorded for 9 and 13 AR days with values of 0.556 and 0.563, respectively. As shown in Fig. 15, the GWL raised with the increase of AR. There is a strong correlation between the GWL and the AR.

In summary, the RWL leading 28 days and 35 days, and the AR leading 9 days and 13 days were chosen as the optimal triggering factors of the GWO-LSTM model.

### Prediction results and analysis

Seven hundred and seventy-eight measured data from January 19, 2015, to March 6, 2017, were adopted to establish the predicted model. Six hundred and twenty-two data from January 19, 2015 to October 1, 2016 were selected as the training samples. The following 78 groups of data (October 2, 2016–December 18, 2016) were used as the test samples to evaluate the model's accuracy and determine the optimal prediction model. The data from December 19, 2016 to March 6, 2017 were used as a validation set to verify the generalization ability of the optimal prediction model. The optimal hyperparameters of the LSTM model were obtained by using the training set and the testing set. Then, the training set and the testing set were combined into the training set of the validation set. The maximum and minimum values of input and output for normalization and inverse normalization were obtained respectively from the training data set to prevent information leakage. Each triggering factor was normalized to [− 1, 1] using linear normalization:

11 $$x^{ * } = 2 \times \frac{{x - x_{\min } }}{{x_{\max } - x_{\min } }} - 1$$

where *x ∗ *is the normalized value, *x* is the original value, *x*_*max*_ is the maximum value of the samples, and *x*_*min*_ is the minimum value of the samples. Based on an original program written in Matlab R2020b for this specific analysis, the LSTM model and the SVR model were used to learn and train the data in the training samples. When the network error converged to the expected value, the network was used to predict the data in the test samples to test the generalization ability. The single-factor GWO-LSTM model, the MIC-GWO-LSTM model, and the MIC-GWO-SVR model were proposed in this paper to compare and analyze the prediction performance of different models.

As for the single-factor GWO-LSTM model parameter setting, an LSTM model with *n*_*h*_ (number of hidden units) layer, *n*_*f*_ (number of triggering factors) input nodes, and one output node was constructed. Triggering factors were not considered when applying the single-factor GWO-LSTM model. The LSTM model selected the sigmoid function as the activation function. The PredictAndUpdateState function trained the recurrent neural network and updated the network state. The number of triggering factors *n*_*f*_ was set as 1. The hyperparameters of the LSTM model include the number of hidden units *n*_*h*_, the max epochs *n*_*E*_, and the initial learning rate *l*_*r*_. In the GWO algorithm parameter setting, the number of hidden units *n*_*h*_ = [0, 200], the max epochs *n*_*E*_ = [0, 200], the initial learning rate *l*_*r*_ = [0.001, 0.5] were set. The GWO algorithm searched for the optimal hyperparameters through the test set; the number of grey wolf groups and generations was 30, 50 respectively. The *RMSE* between the measured and predicted values was set as the fitness function.

As for the MIC-GWO-LSTM model parameter setting, when the MIC-GWO-LSTM model was established, triggering factors that had greater influence on the GWL were selected as input variables. The GWL was selected as the output variable. The parameter *n*_*h*_ was determined according to the number of triggering factors. In the GWO algorithm parameter setting, the number of hidden units *n*_*h*_ = [0, 200], the max epochs *n*_*E*_ = [0, 200], the initial learning rate *l*_*r*_ = [0.001, 0.5] were fixed. The number of grey wolf groups and generations were set as 30, 50 respectively.

As for the MIC-GWO-SVR model parameter setting, the MIC-GWO-SVR model was proposed to make a comparative analysis with the single-factor GWO-LSTM model and MIC-GWO-LSTM model. Triggering factors of the MIC-GWO-SVR model input were the same as the MIC-GWO-LSTM model. The hyperparameters of the SVR model include the penalty factor *C* and the radial basis kernel function *γ*^36^. In the GWO algorithm parameter setting, the penalty factor *C* = [0,100], the kernel function parameter is *γ* = [0, 100], the principal component is set to 95%, and the cross-validation value is *v* = 5. The number of grey wolf groups and generations were set as 30, 50 respectively. The *RMSE* between the measured and predicted values was set as the fitness function.

**Figure 16:**
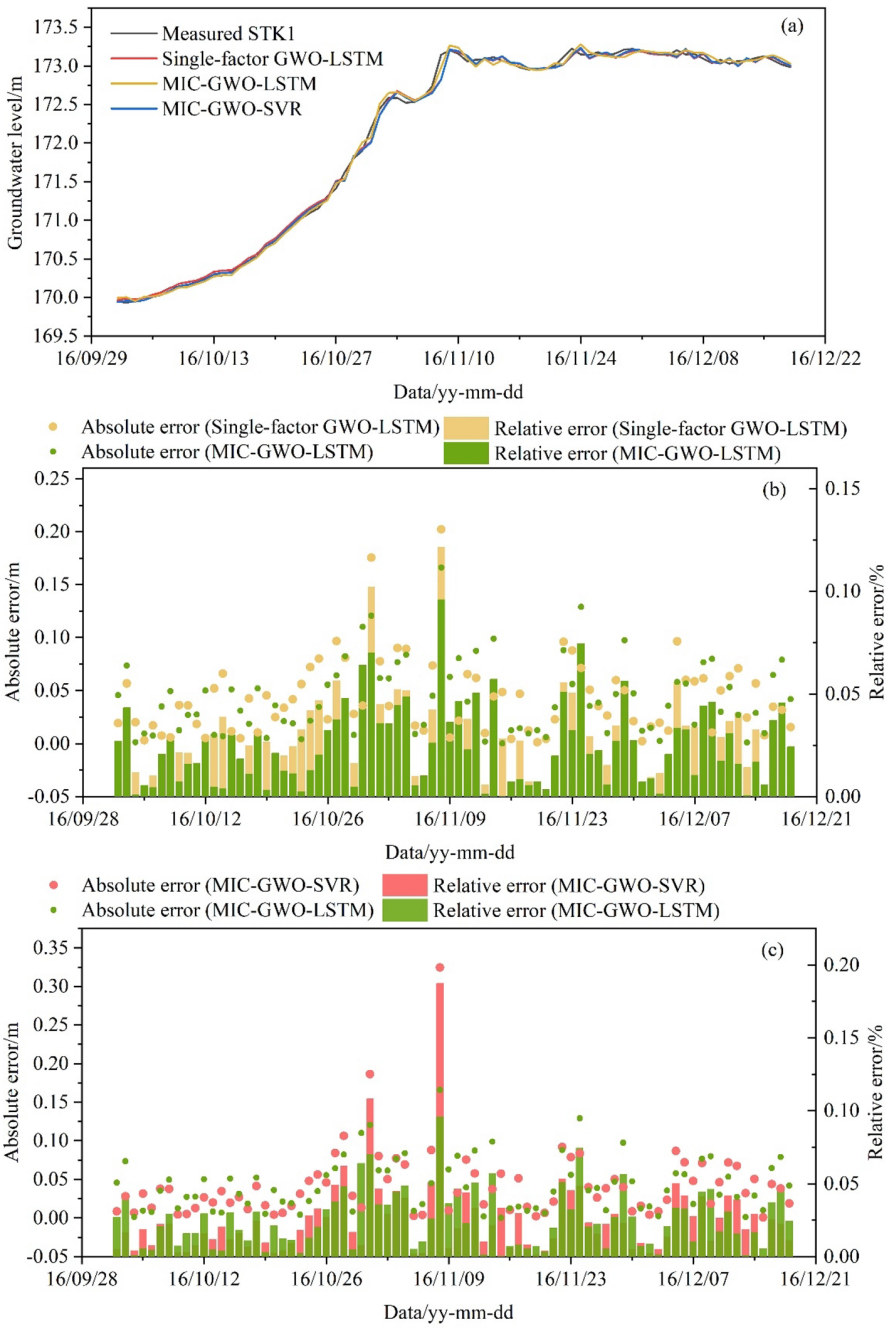
Prediction result of the test set. (**a**) Direct prediction of the GWL by the LSTM and SVR models, (**b**) the absolute error and relative error of the Single-factor GWO-LSTM model and the MIC-GWO-LSTM model, (**c**) the absolute error and relative error of the MIC-GWO-SVR model and the MIC-GWO-LSTM model.

Prediction results of the test set, using the GWO algorithm to search for optimal hyperparameters, are shown in Table 2. The three optimized models show good prediction ability on the test set (Fig. 16a). The *RMSE* and *R*^2^ of the single-factor GWO-LSTM model were 0.0624 and 0.9974. The *RMSE* and *R*^2^ of the multi-factor GWO-SVR model were 0.0615 and 0.9975, whereas the values of the MIC-GWO-LSTM model were 0.0544 and 0.9977, respectively. The MIC-GWO-LSTM model has a better training ability than the MIC-GWO-SVR and the single-factor GWO-LSTM model. The most significant errors for the single-factor GWO-LSTM (Relative error 0.125%), MIC-GWO-SVR (Relative error 0.187%), and MIC-GWO-LSTM (Relative error 0.096%) models for the direct prediction of the GWL occurred on November 8 (Fig. 16b,c). This time point is the inflection point when the RWL rose from 145 to 175 m. It shows that these prediction models have a certain prediction delay for the inflection point of data.

**Table 2:** Prediction results of the different prediction model.

Model	Hyperparameters	The test set		The validation set	
		*RMSE*	*R*^2^	*RMSE*	*R*^2^
Single-factor GWO-LSTM	*n*_*h*_ = 2, *n*_*E*_ = 88, *l*_*r*_ = 0.062	0.0624	0.9974	0.0660	0.9641
MIC-GWO-LSTM	*n*_*h*_ = 47, *n*_*E*_ = 169, *l*_*r*_ = 0.002	0.0544	0.9977	0.0457	0.9830
MIC-GWO-SVR	*C* = 7.212, *γ* = 0.235	0.0615	0.9975	0.0532	0.9769

**Figure 17:**
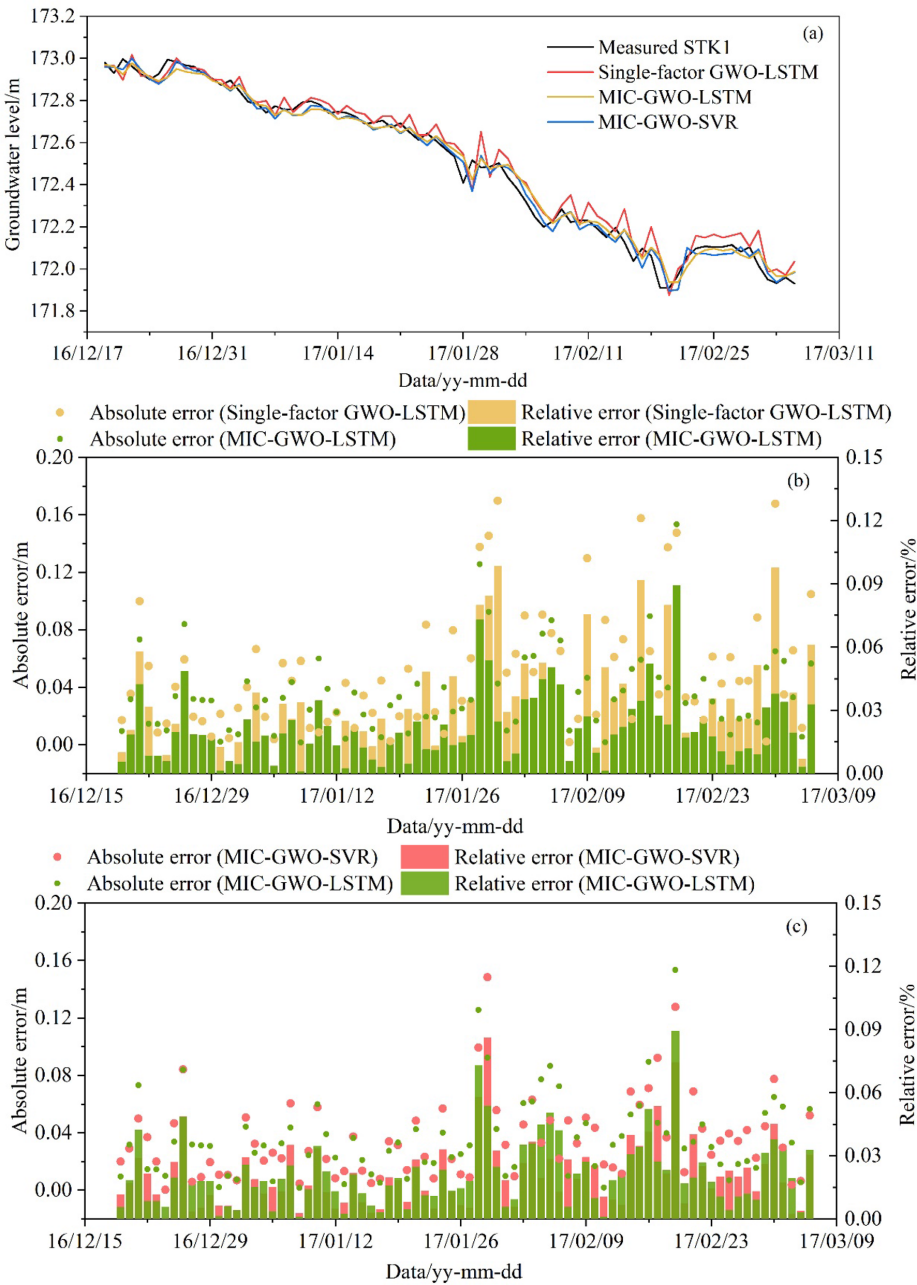
Prediction result of the validation set. (**a**) Direct prediction of the GWL by the LSTM and SVR models, (**b**) the absolute error and relative error of the Single-factor GWO-LSTM model and the MIC-GWO-LSTM model, (**c**) the absolute error and relative error of the MIC-GWO-SVR model and the MIC-GWO-LSTM model.

The optimized hyperparameters (Table 2) were used to predict the validation set. Figure 17a shows that the predicted results of the three models were all consistent with the trend of measured data. Due to the lack of triggering factors, the single-factor GWO-LSTM model, which represents more fluctuation in the validation set, has poor prediction and generalization ability (*RMSE* = 0.0660, *R*^2^ = 0.9641). The *RMSE* and *R*^2^ of the MIC-GWO-SVR model were 0.0532 and 0.9769, whereas the values of the MIC-GWO-LSTM model were 0.0457 and 0.9830, respectively. The single-factor GWO-LSTM model has higher fluctuations and errors in the whole prediction sequence (Fig. 17b), and has a lower generalization ability than the MIC-GWO-LSTM model. The MIC-GWO-SVR model and the MIC-GWO-LSTM model have different prediction abilities and represent different prediction errors on the validation set (Fig. 17c). They represent the complicated nonlinear relationship between the GWL and its triggering factors and have good generalization ability on the validation set. In general, the MIC-GWO-LSTM model represents a higher prediction accuracy than the MIC-GWO-SVR model.

## Discussion

**Figure 18:**
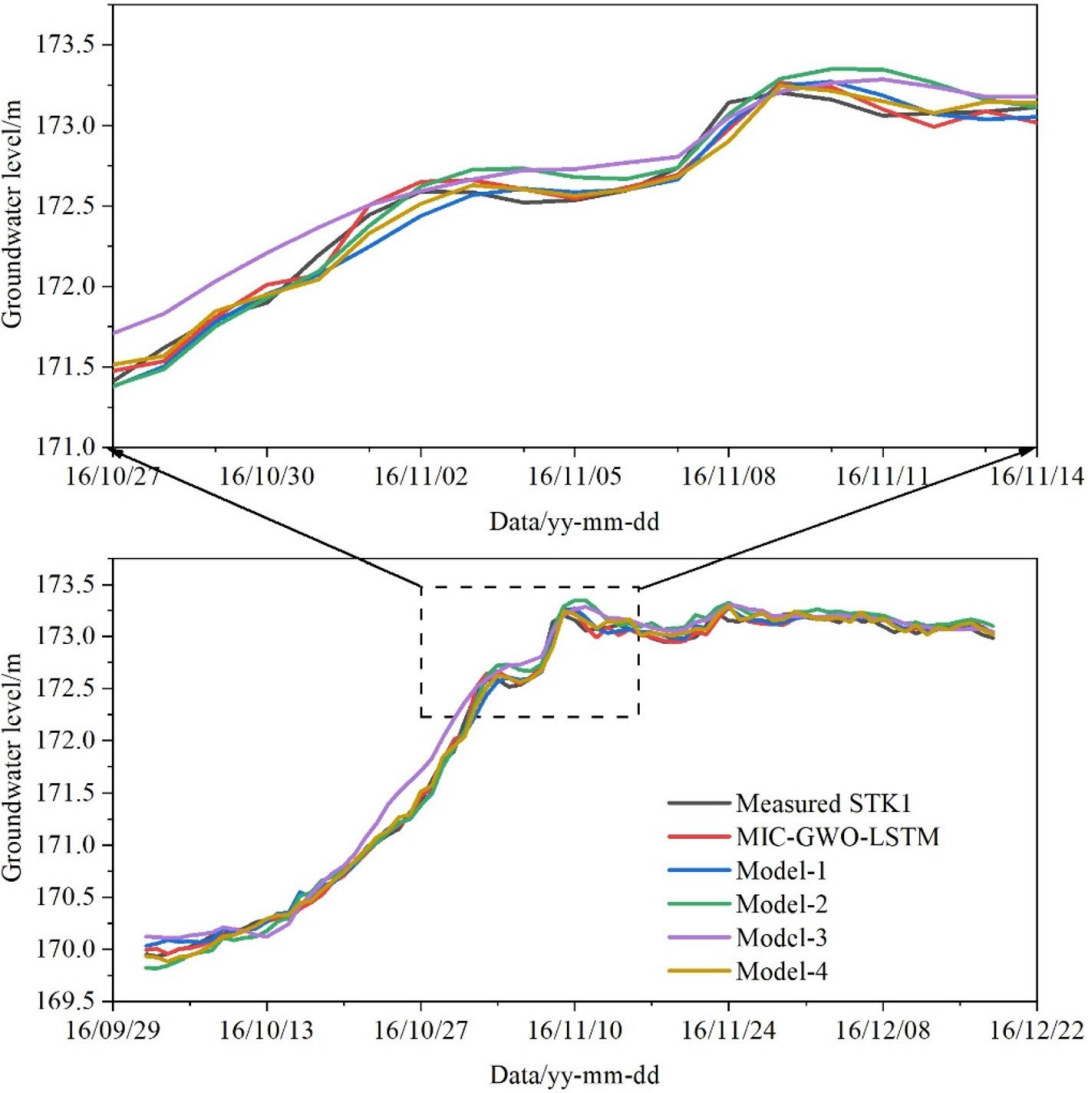
Prediction results under different triggering factors of the test set.

**Figure 19:**
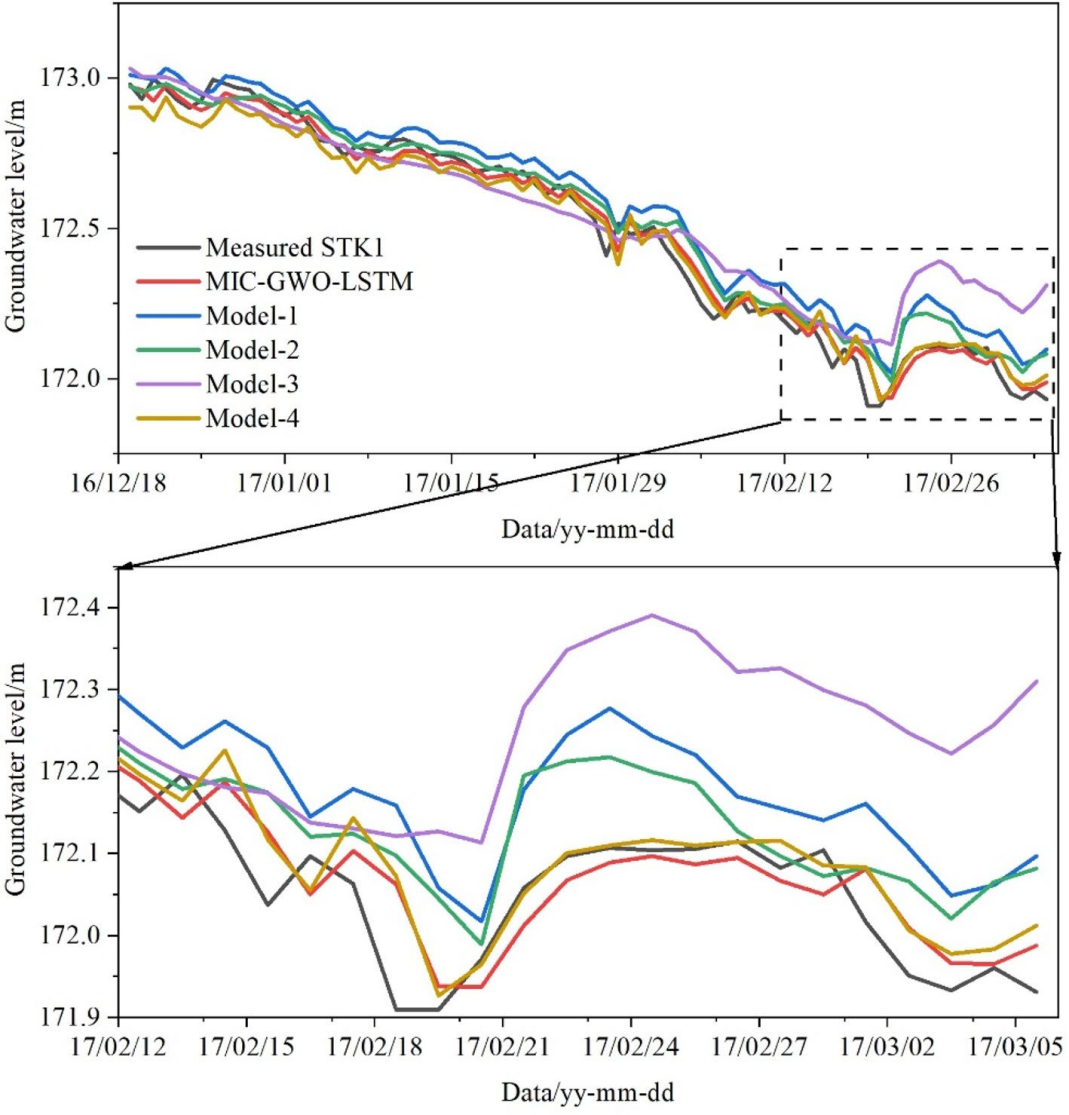
Prediction results under different triggering factors of the validation set.

The accurate prediction of the GWL needs to find the main control factors^55^. The AR and the RWL are the two main factors that can change the hydrological conditions of the GWL in the TGRA. Four sets of prediction experiments were proposed with different triggering factors to compare the MIC-based feature engineering's prediction stability. As shown in Table 3, models of four different triggering factors were established and compared with the MIC-GWO-LSTM model. They all have good prediction ability in the training set, especially in the stage of groundwater level fluctuation from October 27 to November 14 (Fig. 18), mainly as the GWO algorithm searched the suitable hyperparameter of the LSTM model under specific prediction conditions. The prediction results of the four models are quite different in the validation set (Fig. 19). The prediction accuracy and generalization ability of the MIC-GWO-LSTM model decreased with input factors. These triggering factors contain a lot of duplicates and redundant information because they are mainly calculated by the measured data of the current day. Two factors (model-4) with high correlation were screened out by the Pearson correlation coefficient (PCC) algorithm. The model-4 also predict the GWL with high prediction and generalization ability. The redundant triggering factors will reduce the prediction accuracy. The length of input days is not the only criterion for input factors, and the interaction of different factors should also be considered. Krakac^4,26^ selected many triggering factors to predict groundwater depth, including 0–100-day antecedent precipitation and 0–100-day evapotranspiration. This method only focuses on the data itself and ignores the natural phenomena. More input data will increase the operation time and not guarantee prediction accuracy. Cao^12^ applied the grey correlation model to calculate the correlation degree between groundwater and triggering factors but ignored the collinearity of redundant features, which may reduce the generalization ability of the prediction model. In this paper, the time lag of the GWL relative to the RWL and the RA was determined by the MIC-based feature engineering, which integrated the factor selection process with the learner training process, and effectively improved the prediction accuracy and the generalization ability of the prediction model. One should consider developing accurate triggering factors for reducing the model uncertainly in the future.

**Table 3:** The prediction accuracy comparisons between different triggering factors of the GWO-LSTM model.

Model	The triggering factors set	Hyperparameters			The test set		The validation set	
		*n*_*h*_	*n*_*E*_	*l*_*r*_	*RMSE*	*R*^2^	*RMSE*	*R*^2^
Model-1	RWL 1–7 days leading, AR 1–7 days	132	190	0.009	0.0668	0.9970	0.0703	0.9597
Model-2	RWL 1–14 days leading, AR 1–14 days	14	180	0.007	0.0675	0.9970	0.0706	0.9594
Model-3	RWL 1–28 days leading, AR 1–28 days	14	107	0.001	0.1289	0.9889	0.1370	0.8470
Model-4	RWL 28 days leading, AR 7 days (The PCC algorithm)	14	79	0.049	0.0586	0.9977	0.0575	0.9731

The LSTM model saves and uses the historical information and gives full play to extract correlation information^45^. It also represents a good prediction result in the situation of enough samples. The main control factor of the LSTM model is the hyperparameters. Compared with the grid search, the GA, and the PSO algorithms, the GWO algorithm avoids falling into local optima in high-dimensional and has a lower convergence speed in the iterative process. In this paper, The GWO-LSTM model has better generalization performance and improves the prediction accuracy of the GWL by searching for the hyperparameters of the LSTM model.

The fluctuation of the GWL is a complex dynamic system, which is related to hydrological and geological conditions. In the long-term observation period, the effect of periodicity and randomness is reflected in the GWL measured data. The GWL is also closely related to the slope structure and macroscopic deformation characteristics (earth cracks, etc.). Earth cracks increase the permeability of the soil and make groundwater flow more frequently^12^. How to predict the GWL by combining the characteristics of meteorology, hydrology, landslide mass, and landslide macro deformation needs further research.

Compared with the RA and the RWL, the GWL can directly reflect the hydrological characteristics of landslides. The landslide velocity changed with the fluctuation of the GWL because it causes the change of pore water pressure in the soil and directly affects the deformation characteristics^10^. Therefore, a more practical GWL prediction is helpful to predict landslide displacements, which could be valuably exploited to establish a landslide early warning system.

## Conclusion

The monitoring and prediction of the GWL are essential for establishing a landslide early warning system. A MIC-GWO-LSTM model for predicting the GWL of the landslide was tested. The conclusions are summarized below:

1. There is a specific correlation in time series between the GWL with the RA and the RWL. The influence of RWL on the Sifangbei landslide is mainly concentrated on the front area. The GWL is mainly affected by rainfall as the distance from the Yangtze River increases. The farmland and pond also affect the change of the GWL.
2. The MIC algorithm effectively evaluates the relationship between factors and the GWL and analyses the main triggering factors of the RWL and the AR, which improved the generalization ability of prediction modeling.
3. The results show that the prediction accuracy of the GWL prediction model based on the MIC-GWO-LSTM model is better than that of other prediction models.

In general, the MIC-GWO-LSTM model proposed in this paper can effectively construct the response relationship between the GWL and triggering factors. Meanwhile, many machine learning algorithms have been developed for landslide susceptibility mapping and risk analysis^27,56^. Thus, the novel method proposed in this paper is recommended for conducting landslide susceptibility mapping, landslide risk analysis, and other fields.

## Data Availability

Restrictions apply to the availability of these data. Data were obtained from the Geo-environmental monitoring office of Wanzhou District and are available from the authors with the permission of the Geo-environmental monitoring office of Wanzhou District.
